# Serum Levels of Trace Elements in Patients with Testicular Cancers

**DOI:** 10.1590/S1677-5538.IBJU.2014.0460

**Published:** 2015

**Authors:** Mehmet Kaba, Necip Pirinççi, Mehmet Bilgehan Yüksel, İlhan Geçit, Mustafa Güneş, Murat Demir, HurremTuran Akkoyun, Halit Demir

**Affiliations:** 1 Department of Urology, Faculty of Medicine, Yuzuncu Yıl University, Van, Turkey; 2 Department of Urology, Faculty of Medicine, Celal Bayar University, Manisa, Turkey; 3 Department of Veterinaryand HealthSciences,Çiçekdağı VocationalCollege, Ahi Evran University, Kırşehir, Turkey; 4 Department of Chemistry, Faculty of Scienceand Art, Yuzuncu Yıl University, Van, Turkey

**Keywords:** Testicular Neoplasms, Trace Elements, Tumor Markers, Biological

## Abstract

**Introduction:**

Trace elements are primary components of biological structures; however, they can be toxic when their concentrations are higher than those needed for biological functions.

**Materials and Methods:**

In the present study serum levels of trace elements were measured in 30 patients (mean age was 26.9±11.2 years) newly diagnosed with germ cell testicular cancer and 32 healthy volunteers (mean age: 27.4±10.8) by using furnace atomic absorption spectrophotometer. Serum samples were stored at-20°C until assays.

**Results:**

In patients with germ cell testicular cancer, the diagnosis was seminoma in 15, mix germ cell tumor in 7, embryonal carcinoma in 4, yolk sac tumor in 2 and teratoma in 2 patients. There was stage I testicular tumor in 19 patients (63.3%) while stage II in 6 patients (20.0%), stage IIIA in 4 patients (13.3%) and stage IIIC in one patient (3.4%). It was found that serum Co, Cu, Mg and Pb levels were increased (p<0.05), whereas Fe, Mn, and Zn levels were decreased in patients with testicular cancer (p<0.05).

**Conclusions:**

These alterations may be important in the pathogenesis of testicular cancers; however, further prospective studies are needed to identify the relationship between testicular cancer and trace elements.

## INTRODUCTION

Testicular cancers are the most commonly seen cancers in men aged 15-40 years (1). They comprise 1% of all cancers in men. Germ cell tumors comprise 90-95% of all primary testicular tumors. In the past, overall survival rate was approximately 10%, while it increased up to 90% by advances in tumor markers and imaging techniques, and cisplatin-based chemotherapy regimens. The incidence of testicular cancer varies according to race and socioeconomic status. The likelihood of incident testicular cancer is higher in Scandinavian countries, while it is lower in Asian countries (2).

The trace elements are primary components of biological structures; however, they can exert toxic effect when their concentrations are higher than those needed for biological functions. Moreover, the toxicity can also be true for other non-essential elements which have very similar atomic properties and ability to mimic the reactivity of trace elements. Thus, this above-mentioned toxicity/duality leads biological system to develop ability of recognizing and delivering the metal to its target without enabling it to involve in toxic reactions (3, 4). Proteins are the primary compounds involved in this recognition and transport. The majority of the associations between a trace element and other biological molecules result in undesirable chemical modifications in these molecules.

In living organisms, biological mechanisms have been developed in order to use vital trace elements including zinc and copper and to minimize toxic effects of heavy metals such as cadmium, mercury and lead (5). Oxidative processes are most intensive in a background where an imbalance is present in trace elements involved in the structure of enzymes accounted for antioxidant defense (6). Alterations in the ion content of trace elements including iron, copper and zinc can affect activity of antioxidants (7). In several cancers, it has been reported that significant differences occur in the normal distribution of iron, copper and zinc (8). In some trials, serum Cu/Zn ratio was employed as chemoprophylaxis (9). Moreover, it has been also used to evaluate and assess the prognosis in patients with cancer (10, 11).

In the present study, serum levels of trace elements were measured and it was found that Mn, Co, Cu, Mg, Fe and Zn concentrations were altered in sera of the patients with testicular cancer in comparison to healthy subjects.

## MATERIALS AND METHODS

The study included 30 men with germ cell testicular cancer. Mean age was 26.9±11.2 years. All patients were lifetime non-smokers and had no history of alcohol addiction, drug abuse, antioxidant use and metabolic diseases. No comorbid disease was present in any of the patients. All patients had newly diagnosed testicular cancer and preoperative blood samples were taken. Thirty two healthy male subjects (mean age: 27.4±10.8) were randomly selected as controls among volunteers who had no history of smoking, alcohol consumption, drug or antioxidant use, and known comorbid disease. The patient and control groups had similar socioeconomic status.

According to 2009 TNM tumor staging system, there was stage I testicular tumor in 19 patients (63.3%) while stage II in 6 patients (20.0%), stage IIIA in 4 patients (13.3%) and stage IIIC in one patient (3.4%). The study was conducted in accordance to Helsinki Declaration, 1989 Revision. All participants gave written informed consent before participation in the study.

### 

#### Blood Samples

Blood samples were drawn at morning after 12 hours fasting and stored on ice at 4°C. Then, sera were separated by centrifugation at 3000 rpm for 10 minutes. Serum samples were stored at −20°C until assays.

#### Measurements of Mineral-Heavy Metal Levels

Two milliliters of HNO3/H2O2 mixture (2:1) were added to 0.7 g of the serum samples. The mixture was placed into the water bath at 70°C for 30 min and stirred occasionally. Then, one mL of the same acid mixture was added, and the mixture was transferred into a Teflon vessel bomb for the microwave oven. The bomb was closed, and the solution was placed inside the microwave oven. Radiation was applied for 3 min at 450 W. After addition of 0.5mL of the same acid mixture, radiation was repeated for 3 min. After cooling for 5 min, 2.0mL of 0.1mol/L HNO^3^ was added, and the solution was transferred to a Pyrex tube. After centrifugation, the clear solution was used to determine Mn, Cd, Cu, Pb, Fe, Mg, Co and Zn levels. They were measured by using atomic absorption spectrophotometer technique with a UNICAM-929 spectrophotometer (Unicam Ltd, York Street, Cambridge, UK).

### Statistical Analysis

All data were analyzed by using SPSS for Windows version 13.0. Descriptive statistics of the traits evaluated were expressed as mean, standard deviation, minimum and maximum values. Mann-Whitney U test was used for intergroup comparisons. P<0.05 was considered as significant.

## RESULTS

There was seminoma in 15, mix germ cell tumor in 7, embryonal carcinoma in 4, yolk sac tumor in 2 and teratoma in 2 patients. The demographic characteristics are presented Table-1. Trace elements levels are presented in Table-2. No statistical difference was detected between patients with testicular cancer and controls regarding age and body mass index (BMI).

**Table 1:** Demographic characteristics.

Demographic characteristics	Patients (n = 30)	Control (n = 32)	p Value
Age (years) (mean±SD)	26.9±11.2	27.4±10.8	>0.05
BMI (kg/m^2^)	27.0±2.7	28.0±2.1	>0.05

**Table 2:** Descriptive Statistics and Comparison Results According to the Groups for specifications.

		N	Mean	Std. Dev.	Min.	Max.	p
Cd	Patient	30	0.008636	0.020831	0.00194	0.07781	0.083
	control	32	0.001109	0.000114	0.00101	0.00135	
Co	Patient	30	0.002506	0.001108	0.00107	0.00432	0.001
	control	32	0.001102	0.000986	0.00101	0.00143	
Cu	Patient	30	1.0944	0.3992	0.01	1.84	0.001
	control	32	0.84	0.13474	0.52	0.98	
Fe	Patient	30	0.917971	0.404285	0.0433	1.588	0.001
	control	32	2.430144	0.189439	2.0121	2.7998	
Mg	Patient	30	24.4	2.88236	15.83	28.47	0.001
	control	32	0.7527	0.18918	0.11	0.99	
Mn	Patient	30	0.010167	0.008136	0.00145	0.03986	0.001
	control	32	0.7527	0.189182	0.11114	0.98991	
Pb	Patient	30	0.035	0.019655	0.00276	0.07757	0.001
	control	32	0.001204	0.000131	0.001	0.0015	
Zn	Patient	30	0.90998	0.330196	0.036	1.72	0.001
	control	32	2.96368	0.443655	2.125	4.021	

Trace element levels were compared between groups. It was found that Co, Cu, Mg and Pb levels were significantly higher in the patient group compared to the controls. It was also found that Zn, Fe and Mn levels were significantly lower in the patient group compared to controls. Cd levels were found to be higher in patients with testicular tumors compared to controls, but this didn't reach statistical significance.

## DISCUSSION

Traces elements are accepted as essential components of biological structures, which play complex roles in the cancer development or inhibition. There are many questions regarding their essential and toxic effects on human health above the concentrations needed for their biological functions. In this context, there are conflicting results in the literature (12, 13).

Mn is an essential element which is required for several enzyme activities. It has a major role in the antioxidant defense system and comprises part of SOD enzyme (14). Therefore, it can be suggested that decreased serum Mn concentrations with disturbance of antioxidant mechanism can make target organs sensitive to carcinogens. Decreased serum Mn concentrations were reported in patients with bladder and renal cancers (15, 16). Our study showed that serum Mn concentrations were lower in patients with testicular cancer compared to controls. According to our hypothesis, Mn can result in oxidant/antioxidant imbalance in patients with testicular cancer, as being a trace element affecting oxidation status.

Zinc plays an anti-carcinogenic role through structural stabilization of deoxyribonucleic acid (DNA), ribonucleic acid (RNA), and ribosome. Zinc is also important in the functions of several transcription factors and proteins that are involved in the recognition of specific DNA sequences and regulation of gene transcription. Zinc has a protective effect against free-radical injury (17). In previous studies, it has been reported that serum Zn concentrations were decreased in patients with ovarian, cervical, bladder and renal cancer (15–19). It seems that it is necessary to determine serum zinc levels in patients with ovarian cancer in comparison with healthy controls in order to fully elucidate the relationship between serum Zn levels and ovarian cancer. In addition, in a previous study, there were normal or elevated zinc concentrations only in the sera of the cases with primary liver cancer in contrast to decreased serum zinc levels in remaining cases, suggesting a differential sign favoring transformation from hepatocirrhosis to cancer (20). Our study demonstrated decreased Zn concentrations in patients with testicular cancer compared to controls.

Fe is a trace element which is physiologically essential but dangerous in biochemical aspect. Either excess or deficiency of Fe can lead to oxidative DNA damage, although it is an important nutritional element (21). Moreover, it was proposed that low iron levels may have a role in the prevention of infection and cancer (22).

There is substantial evidence supporting the hypothesis of a relationship between testicular cancer and disruption of iron metabolism and that testicular cancer may be a clinical manifestation of iron-induced testicular damage, presumably through an underlying free-radical mechanism (23). In our study, results indicated that there was a significant difference in serum Fe levels between patient and control groups.

Physiological Cd doses lead to an increased endothelial permeability through inhibition of endothelial proliferation and induction of cell death mediated by DNA damage, which is also inhibited by zinc (24). It has been reported that doubling of soil cadmium is associated with an increased risk for lung cancer by 57% (25). It was also shown that serum Cd levels were increased in patients with bladder cancer (3). In cadmium carcinogenicity, proto-oncogene activation, tumor suppressor gene inactivation, disrupted cell adhesion and inhibition of DNA repair are the implied cellular and molecular mechanisms (26, 27). In addition, it has been reported that elevated Cd concentration may result in prostate, kidney and lung cancers (28). In the past decade, it has been reported that the injection of cadmium metal powder and various cadmium compounds induce sarcoma in local and interstitial cell testicular tumor in a systemic manner (29). In our study, Cd levels were found to be higher in patients with testicular cancer compared to controls. However, the difference didn't reach statistical significance.

In our study, it was also demonstrated that serum levels of Pb, Co, Cu and Mg were increased when compared to controls. It is well-known that Pb and Cd are toxic and carcinogenic metals (25). It has been suggested that Pb has a predisposing role in the carcinogenesis with inhibition of DNA synthesis and repair, oxidative injury and interaction with DNA-binding proteins and tumor suppressor proteins (30, 31). Exposure to inorganic lead at early life induces teratoma and pre-neoplasm of renal and urinary bladder (32). Recent research indicated negative health consequences of lower level of lead exposure, including impaired functions of renal tubular cells, inhibition of sperm formation, fetal damage, decelerated velocity of motor nerves, and central nervous system dysfunction as well as hypertension and other cardiovascular diseases (33, 34). It was also shown that Pb levels were significantly increased in patients with malignant glioma compared to controls (35). In our study, Pb levels were found to be significantly higher in the patient group compared to controls.

Co is a constituent of vitamin B12 in humans. In human studies, evidence is inconclusive regarding relationship between inhalational exposure to cobalt and cancer. In the only available study on oral exposure to cobalt, no correlation was reported between cobalt and cancer deaths. In a study on workers refining and processing cobalt and sodium, it was shown that there was an increase in deaths from lung cancer in workers exposed to cobalt. Effects of exposure to cobalt salts were investigated in only one study. Preliminary results indicated an increased risk for lung cancer in those working at cobalt production, but follow-up didn't show any increase in the risk for cancer (36). However, that study was limited by the small number of workers who developed cancer. In our study, Co levels were found to be significantly higher in the patient group compared to controls.

Cu plays a role in the production of hemoglobin, myelin, collagen and melanin as an essential nutrient (4). In recent studies, it was shown that normal immune function requires adequate Cu intake (3, 4). It is well-known that serum Cu levels increase in several malignancies such as osteosarcoma, gastrointestinal tumors and lung cancer (37). In some studies, it was shown that there was an increase in Cu levels within tumor cells or lung epithelial lining fluid of patients with lung cancer (38, 39). In our study, serum Cu levels were found to be significantly higher in the patient group compared to controls.

The observed effects of magnesium on either tumor transplant-or chemical-induced cancers depended on the duration of Mg supplementation or deficiency (40). Optimal Mg supplementation may have prophylactic effects against some neoplasms, but Mg alone isn't recommended for therapeutic purposes as cancer cells have also high metabolic requirements (40). Recently, in rats, it was shown that Mg supplementation inhibited increased DNA synthesis at colon epithelium. In that study, it was suggested that the finding might be related to suppression of oncogene-induced bowel carcinogenesis by Mg (41). In our study, serum Mg levels were found to be significantly higher in patients with testicular cancer compared to controls.

In our study, serum levels of trace elements were assessed in germ cell testicular cancer. Serum levels of trace elements can differ in different germ cell testicular cancer as in serum levels of tumor markers. Further studies are needed to evaluate this issue. Germ cell testicular cancers (other than choriocarcinoma) initially spread to retroperitoneal lymph nodes. Retrospective studies with larger series which compare early stage, non-metastatic and metastatic tumors are necessary in order to investigate effects of metastasis on alterations in serum trace element levels.

In conclusion, an association was observed between testicular cancer and trace elements in the present study. We also think that increases in Co, Cu, Mg and Pb levels and decreases in Zn, Mn and Fe levels can play important roles in the induction of testicular cancers. However, future prospective studies on the reasons of alteration in the serum concentrations of trace elements in patients with testicular cancer seem to be well grounded. Further prospective studies are needed to clarify the relationship between various stages of testicular cancer and serum levels of trace elements.
